# Mutational Analysis of Colistin-Resistant *Pseudomonas aeruginosa* Isolates: From Genomic Background to Antibiotic Resistance

**DOI:** 10.3390/pathogens14040387

**Published:** 2025-04-15

**Authors:** Telma De Sousa, Hsin-Yao Wang, Ting-Wei Lin, Manuela Caniça, Miguel J. N. Ramos, Daniela Santos, Catarina Silva, Sónia Saraiva, Racha Beyrouthy, Richard Bonnet, Michel Hébraud, Gilberto Igrejas, Patrícia Poeta

**Affiliations:** 1 MicroART—Antibiotic Resistance Team, Department of Veterinary Sciences, University of Trás-os-Montes and Alto Douro, 5000-801 Vila Real, Portugal; telmaslsousa@hotmail.com (T.D.S.); xusilva2002@gmail.com (C.S.); soniasaraiva@utad.pt (S.S.); 2 Department of Genetics and Biotechnology, University of Trás-os-Montes and Alto Douro, 5000-801 Vila Real, Portugal; gigrejas@utad.pt; 3 Functional Genomics and Proteomics Unit, University of Trás-os-Montes and Alto Douro, 5000-801 Vila Real, Portugal; 4 Associated Laboratory for Green Chemistry, University NOVA of Lisbon, 1099-085 Caparica, Portugal; 5 Department of Laboratory Medicine, Chang Gung Memorial Hospital at Linkou, Taoyuan 333, Taiwan; mdhsinyaowang@gmail.com (H.-Y.W.); weitinglin66@gmail.com (T.-W.L.); 6 School of Medicine, National Tsing Hua University, Hsinchu 300, Taiwan; 7 National Reference Laboratory of Antibiotic Resistance and Healthcare Associated Infections, Department of Infectious Diseases, National Institute of Health Dr. Ricardo Jorge, 1649-016 Lisbon, Portugal; manuela.canica@insa.min-saude.pt (M.C.); miguel.ramos@insa.min-saude.pt (M.J.N.R.); daniela.santos@insa.min-saude.pt (D.S.); 8 Centre for the Studies of Animal Science (CECA), Institute of Agrarian and Agri-Food Sciences and Technologies, University of Porto, 4099-002 Porto, Portugal; 9 CECAV—Veterinary and Animal Research Centre, University of Trás-os-Montes and Alto Douro, 5000-801 Vila Real, Portugal; 10 Institut National de la Santé et de la Recherche Médicale, (UMR1071), Institute National de la Recherche Agronomique (USC-2018), Université Clermont Auvergne, 63000 Clermont-Ferrand, France; rbeyrouthy@chu-clermontferrand.fr (R.B.); rbonnet@chu-clermontferrand.fr (R.B.); 11 Centre National de Référence de la Résistance aux Antibiotiques, Centre Hospitalier Universitaire, 63000 Clermont-Ferrand, France; 12 INRAE, Université Clermont Auvergne, UMR Microbiologie Environnement Digestif Santé (MEDiS), 63122 Saint-Genès-Champanelle, France; michel.hebraud@inrae.fr; 13 Veterinary and Animal Research Centre, Associate Laboratory for Animal and Veterinary Science (AL4AnimalS), University of Trás-os-Montes and Alto Douro, 5000-801 Vila Real, Portugal

**Keywords:** *Pseudomonas aeruginosa*, colistin resistance, WGS, mutations, antibiotic resistance

## Abstract

This study analyzed eleven isolates of colistin-resistant *Pseudomonas aeruginosa*, originating from Portugal and Taiwan, which are associated with various pathologies. The results revealed significant genetic diversity among the isolates, with each exhibiting a distinct genetic profile. A prevalence of sequence type ST235 was observed, characterizing it as a high-risk clone, and serotyping indicated a predominance of type O11, associated with chronic respiratory infections in cystic fibrosis (CF) patients. The phylogenetic analysis demonstrated genetic diversity among the isolates, with distinct clades and complex evolutionary relationships. Additionally, transposable elements such as Tn3 and IS6 were identified in all isolates, highlighting their importance in the mobility of antibiotic resistance genes. An analysis of antimicrobial resistance profiles revealed pan-drug resistance in all isolates, with a high prevalence of genes conferring resistance to β-lactams and aminoglycosides. Furthermore, additional analyses revealed mutations in regulatory networks and specific loci previously implicated in colistin resistance, such as *pmrA*, *cprS*, *phoO*, and others, suggesting a possible contribution to the observed resistant phenotype. This study has a strong impact because it not only reveals the genetic diversity and resistance mechanisms in *P. aeruginosa* but also identifies mutations in regulatory genes associated with colistin resistance.

## 1. Introduction

*Pseudomonas aeruginosa*, a Gram-negative bacterium, holds critical importance in healthcare environments as a leading cause of nosocomial infections, while also posing challenges in treatment due to its resistance to antimicrobial agents and ability to thrive in diverse environmental conditions, causing opportunistic infections in humans, animals, and plants [1]. Infections caused by antibiotic-resistant *P. aeruginosa* are linked to over 300,000 annual fatalities, placing them at the forefront of the World Health Organization’s priority list for the urgent research and development of novel antibiotics [2]. Its capacity to acquire multidrug resistance (MDR) poses a significant concern, as it demonstrates resilience against a wide range of antibiotic categories, such as aminoglycosides (including amikacin, gentamicin, and tobramycin), fluoroquinolones (including ciprofloxacin, ofloxacin, and norfloxacin), carbapenems, and tetracyclines [3]. This escalating risk stems from the pathogen’s remarkable ability to develop resistance via chromosomal mutations, coupled with the rising prevalence of transferable resistance elements.

Colistin, or polymyxin E, is often employed as a final option in treating *P. aeruginosa* infections triggered by isolates (MDR) or extensively resistant (XDR) ones [4]. In *P. aeruginosa*, resistance to colistin can occur either through acquisition or adaptation. In the case of acquired resistance, it primarily arises from the addition of one or two 4-amino-L-arabinose (L-Ara4N) molecules to the 1 and/or 4′ phosphate groups on the lipid A, which serves as the foundation of the lipopolysaccharide (LPS) situated in the outer membrane [5,6]. The biosynthesis and transportation of L-Ara4N molecules are governed by the extensive operon *arnBCADTE* which is intricately regulated by a complex network comprising a minimum of five two-component systems (PmrA/PmrB, PhoP/PhoQ, ParR/ParS, CprR/CprS, and ColR/ColS) [7,8,9,10,11]. The resistance of *P. aeruginosa* to colistin is also recognized for its adaptive nature, characterized by its induction in the presence of the antimicrobial agent and subsequent reversion to the susceptible phenotype in its absence.

Moreover, variations occurring in chromosomal genes responsible for encoding histidine kinase or response regulators within these two-component systems lead to the persistent activation of the *arn* operon. This constitutive activation prompts the continuous synthesis and transportation of L-Ara4N molecules, contributing to the development of resistance to colistin in *P. aeruginosa* [4,12].

Although several mechanisms related to the evolution of colistin resistance have been identified, the explanation for the reversal of colistin-resistant mutants of *P. aeruginosa* to the susceptible phenotype in the absence of antibiotics remains largely limited due to the scarcity of available data [7].

In this study, the main objective was to investigate 11 isolates of colistin-resistant *P. aeruginosa*, originating from two countries, which are associated with different pathologies. To achieve this, various techniques were employed, including the sequencing and analysis of these isolates to identify antibiotic resistance genes that could be involved in this colistin resistance phenotype.

## 2. Materials and Methods

### 2.1. Pseudomonas aeruginosa Isolates

For the isolation of colistin-resistant *P. aeruginosa* from Portugal, seven isolates were obtained from various sample types at the Centro Hospitalar De Trás-Os-Montes E Alto Douro (CHTMAD). Identification was carried out using matrix-assisted laser desorption/ionization coupled to time-of-flight mass spectrometry (MALDI-TOF MS) with BRUKER equipment. Antibiotic susceptibility testing (AST) was performed using the VITEK^®^ 2 COMPACT system (bioMérieux, Marcy-l’Étoile, France) and the Kirby–Bauer disk diffusion method, following the EUCAST (European Committee on Antimicrobial Susceptibility Testing) standards 2022.

In Taiwan, four colistin-resistant *P. aeruginosa* isolates were obtained from the Laboratory of Medicine at Chang Gung Memorial Hospital. AST was conducted using the automated broth microdilution method (Phoenix, BD). The samples were collected over several years from individuals of both sexes, with diverse age ranges and pathological conditions (Table 1).

### 2.2. Evaluation of Antibacterial Activity for Colistin

To determine the minimum inhibitory concentration (MIC) of colistin, the broth microdilution method in Mueller–Hinton broth (Frilabo, Portugal) was employed. After the growth of different isolates for 24 h at 37 °C, the isolates were diluted to a concentration of 5 × 10^5^ cells/mL. Using 96-well microtiter plates, the isolates were exposed to increasing gradients of colistin (up to 512 μg/mL). The MIC was determined after an additional 24 h of incubation at 37 °C. We followed the criteria established by the EUCAST (European Committee on Antimicrobial Susceptibility Testing) 2022, considering isolates with MIC above 2 μg/mL as resistant.

### 2.3. Whole Genome Sequencing

The whole-genome sequencing of Portuguese *P. aeruginosa* isolates (HU121, HU122, HU130, HU134, HW3) was conducted at the Hospital de Clermont-Ferrand, France. DNA extraction was conducted utilizing the DNeasy UltraClean Microbial kit (Qiagen, Hilden, Germany). Following extraction, libraries were prepared employing the Nextera XT Kit sourced from Illumina (San Diego, CA, USA) and underwent sequencing on the Illumina MiSeq system, generating 2 × 300 base pair (bp). Libraries from 1 ng of genomic DNA were prepared using the dual-indexed Nextera XT Illumina library preparation kit before cluster generation and paired-end sequencing (2 × 150 bp) on a NextSeq 550 Illumina platform (Illumina Inc., San Diego, CA, USA). The average depth of the mapped reads stood at 98.4X  ±  9.9 (mean ± standard deviation), with a minimum of 81-fold coverage, while the average breadth of coverage was 95.1% ± 1.0%, referencing the PAO1 genome (NC_002516.2).

The isolates HBSB35 and HU141 were sequenced at the National Institute of Health Dr. Ricardo Jorge in Portugal. Genomic DNA extraction was performed using a MagNA Pure 96 instrument (Roche, Mannheim, Germany) and quantified using Qubit Fluorometric Quantitation (Thermo Fisher Scientific, Waltham, MA, USA).

The sequencing of the *P. aeruginosa* isolates (4098, 2910, B19083-11 and B21097-69) from Taiwan was conducted by the Laboratory of Medicine at Chang Gung Memorial Hospital in Taiwan. DNA extraction was performed using the Qiagen/QIAamp (Hilden, Germany) DNA Mini Kit (250) and quantified using the Qubit 4 Fluorometer with the Qubit dsDNA HS Assay kit. Sequencing was performed on the ONT Nanopore MinION MK1C device using the ONT Ligation Sequencing Kit. Additionally, the NEBNext Ultra™ End Repair/dA-Tailing Module was used for sample preparation.

### 2.4. Assembly and Annotation

De novo assembly was performed using INNUca (v 4.2.2-02) (INNUca GitHub: https://github.com/B-UMMI/INNUca, accessed on 25 May2024), with the following options: speciesExpected “*Pseudomonas aeruginosa*”, genomeSizeExpectedMb “6.3”, runKraken. The Kraken2 database utilized was the standard one, updated as of 5 June 2023.

The samples were deposited in the bioproject PRJNA1082679 in genBank, and they are identified according to Table 1.

Genomic DNA was extracted and sequenced using the Oxford Nanopore Technology (ONT) platform with the R9.4.1 flow cell. Base calling was performed using Guppy v6.1.2 (Oxford Nanopore Technologies) with the super-accurate basecalling model (dna_r9.4.1_450bps_sup.cfg) on a CUDA-enabled GPU to ensure high basecalling accuracy. The raw signal data from the sequencer was converted into nucleotide sequences and stored in FASTQ format for further processing.

De novo genome assembly was conducted using Flye v2.9.1, an assembler specifically optimized for long-read sequencing data. The nano-raw parameter was used to process the uncorrected raw ONT reads, and a minimum overlap of 1000 bp was set to ensure accurate contig formation. The assembly was performed with eight computational threads to optimize processing speed and efficiency. The assembled genome was output to a designated directory for subsequent analyses.

Genome annotation was performed using Prokka, a widely used tool for rapid prokaryotic genome annotation. The annotation pipeline was executed within a Conda-managed environment to ensure reproducibility. The assembled genome in FASTA format was used as the input for Prokka, and annotation was performed with the force option to overwrite any existing output files. The cpus 8 parameter was specified to enable multi-threaded processing, improving computational efficiency. Prokka identified and annotated coding sequences (CDS), rRNA, tRNA, and other genomic features using its curated database, generating standard output files, including GenBank-formatted and GFF3 annotation files for downstream analyses.

### 2.5. Comparative Genomics Analysis

#### 2.5.1. Phylogenomic Classification of Genomes

The construction of the phylogenetic tree, based on SNPs, was facilitated through the employment of the online tool CSI Phylogeny v1.4.

On the other hand, serotyping involves the characterization of bacterial isolates based on specific surface antigens, such as proteins or carbohydrates. The PAst v1.0 tool was developed using the programming language Perl for the in silico serotyping of *P. aeruginosa* isolates using WGS data. It is based on a BLASTn analysis of the assembled input genome, against an OSA cluster database. OSA clusters with >95% coverage in the query genome represent a positive hit for a serogroup [13,14].

The phylogenetic tree was constructed using the MEGA v11.0 [15] and iTol v5.7 (Interactive Tree of Life) tools [16]. The parameter was set as default.

#### 2.5.2. Identification of Antibiotic Resistance Genes and Virulence Genes

The identification of resistance genes was performed according to well-known and curated databases such as ResFinder v4.4.3 [17], the Comprehensive Antibiotic Resistance Database (CARD) [18], and BV-BRC v3.32.31a [19]. The ResFinder v4.4.3. parameters were as follows: 90% threshold for %ID and 60% of minimum length for chromosomal point mutations and acquired antimicrobial resistance genes. CARD and BV-BRC v3.32.31a parameters were set as the default.

The Mobile Element Finder v1.0.3 [20] and IS finder v 2.0 identify mobile genetic elements and their relation to antimicrobial resistance genes. For MobileElementFinder v1.0.3, with the prediction thresholds, an initial analysis with lenient thresholds (coverage = 0.1; sequence identity = 0.1) was conducted. The quality thresholds were defined by inspecting the alignments underlying the predicted composite transposons, IME and unit-transposons with annotated cores and accessory genes. Putative mobile elements based solely on alignments to accessory genes were considered false positives. A mobile element was considered present if the alignment coverage was greater than 90% and the sequence identity was greater than 90%. For IS Finder, the parameters were set as the default.

For the detection of virulence genes, the tools ResFinder v4.4.3 [17] and BV-BRC v3.32.31a [19] were utilized.

#### 2.5.3. Genomic Characteristics of Colistin-Resistant *P. aeruginosa* and Mutation Analysis Based on Literature Reports

Single-nucleotide polymorphisms (SNPs) were identified by mapping filtered reads against the closed reference genome of *P. aeruginosa* PAO1 (NC_002516.2) using Snippy v4.3.6 [21].

Snippy v4.3.6 [21] software is a widely used tool for detecting genetic variants, such as SNPs, deletions, and insertions in bacterial genomes.

For the detection of genome mutations, the tools Snippy v4.3.6 [21] and BV-BRC v3.32.31a [19] were employed. Genomic mutations often play a significant role in determining the presence or absence of antimicrobial resistance. By analyzing genetic mutations using these tools, it is possible to identify changes in the genetic material that may be associated with antimicrobial resistance. The Snippy v4.3.6 tool uses the following default settings: average insert size for paired-end reads (default: 300), minimum coverage depth to report an SNP (default: 10), minimum proportion of reads to call an SNP (default: 0.9), minimum base quality to count a base (default: 20), minimum mapping quality to use a read (default: 60), and minimum base quality to use a base (default: 13).

The tool Clustal Omega v2 [22] was used to align two sequences of interest. The HW3 strain against gb|AAG06466.1|+|cprS, which is the reference strain in the Comprehensive Antibiotic Resistance Database.

## 3. Results

### 3.1. P. aeruginosa Within-Strain Diversity

The 11 isolates analyzed in this study were collected from a variety of sources, with samples from urinary tract infections (UTIs) being predominant. Each of these isolates exhibited distinct sequence profiles (STs), with the isolate B21097-69 identified as sequence type 235 (ST235), characterizing it as a high-risk clone (Table 2). Genetic analyses of the OSA cluster revealed that the predominant serotype is O11, which was identified in four of the isolates. The serotypes O6 and O12 were identified in two of the isolates.

**Figure 1:**
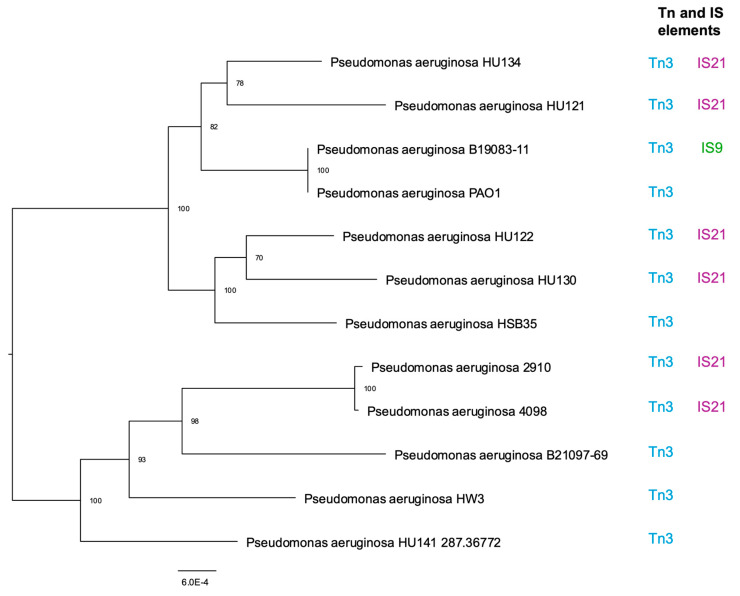
Phylogenetic tree of various *P. aeruginosa* isolates. The tree illustrates the genetic relationships and divergence among the isolates, with bootstrap values indicating the level of statistical support for each branch. High bootstrap values reflect strong confidence in the specific branching points. Some of the Tn and IS elements were represented for each strain. SNPs were identified by mapping filtered reads against the reference genome *P. aeruginosa* PAO1 (NC_002516.2) using Snippy v4.3.6, with default parameters, including a minimum coverage of 10× and a minimum proportion of 0.9 to call a SNP. The Mobile Element Finder v1.0.3 and IS Finder detected mobile genetic elements associated with antimicrobial resistance, considering an element present when sequence coverage and identity exceeded 90%.

The phylogenetic analysis of *P. aeruginosa* isolates reveals significant genetic diversity (Figure 1). The tree shows two main clades. The first clade includes only strain HU141, indicating a distinct evolutionary divergence. The second clade splits into two subclades: one containing strain HW3 and another with additional subgroups.

Notable subgroups include *P. aeruginosa* 2910 and 4098 (bootstrap 100) and HU122 and HU130 (bootstrap 70). A subclade with 82 bootstrap supports includes B19083-11 and PAO1 (bootstrap 100), connected to HU134 and HU121 (bootstrap 78). Bootstrap values reflect the confidence in evolutionary divisions.

Therefore, the phylogenetic tree clearly reveals the genetic diversity relationships among the different isolates of *P. aeruginosa*, with several divisions well-supported by high bootstrap values. Each branch represents a point of evolutionary divergence, helping us to understand how these isolates are related to each other.

### 3.2. Tn and IS Elements

The presence of transposable elements including Tn3, IS6, IS3, IS5, and IS21 was identified in various bacterial isolates.

Tn3, a 4957-base pair transposon, was detected in all studied isolates with notably high prevalence. This transposon encodes three proteins: β-lactamase, which is responsible for resistance to β-lactam antibiotics; a transposase (encoded by the *tnpA* gene); and a resolvase (encoded by the *tnpR* gene).

In addition to Tn3, several IS elements were identified, including IS6. The isolate B19083-11 was the only one to exhibit IS29 (Supplementary Figure S1). In specific isolates, such as HU121, HU122, HU130, HU134, 2910, and 4098, IS21 was identified with high prevalence.

Mobile genetic elements IS21, IS6, and IS9 were detected in the genome of *P. aeruginosa.* They play a crucial role in genetic mobility by facilitating the insertion, deletion and rearrangement of genes, including those associated with antibiotic resistance and environmental adaptation.

### 3.3. Geno-Phenotype in Antibiotic Resistance

The comparative analysis of resistance to different antipseudomonal agents is shown in Table 3. It is noted that the examined isolates display resistance to a high number of antibiotic classes, so they are characterized by being extensively drug resistant (XDR).

The genomes of these isolates harbor several genes associated with resistance to β-lactams, aminoglycosides, quinolones, phenicols, sulfonamides, and colistin (Table 4).

The predominance of resistance genes to the β-lactam class is particularly high in all isolates, with class D (Oxacillinase β-lactamases) standing out; these have a serine-based structure. The *bla_OXA-396_* gene is the most prevalent, found in four of the analyzed isolates, followed by *bla_OXA-494_*, detected in three isolates. Other genes such as *bla_OXA-846_*, *bla_OXA-488_*, *bla_OXA-848_*, *bla_OXA-395_*, and *bla_OXA-903_* were detected in two of the examined isolates.

The β-lactamases of class A have a structure based on serine, similar to classes C and D. Most of the genes responsible for encoding these enzymes are located on plasmids, which implies the possibility of transfer between bacteria, although there are exceptions where such genes are found on the bacterial chromosome. Within this class belong the extended-spectrum β-lactamases (ESBL); as indicated by the name, they have a significantly broader spectrum of inhibition compared to penicillinases. They are capable of hydrolyzing all penicillins, including those associated with β-lactamase inhibitors, as well as almost all cephalosporins and Aztreonam. Strain B19083-11 is the only one to contain the *bla_SHV-12_* gene.

Unlike other classes, class B has a metal-based structure, referred to as metallo-β-lactamases (MBL). The *bla_VIM-3_* gene was detected in isolates 4098 and 2910. *P. aeruginosa* expressing MBL is resistant to all penicillins, all cephalosporins, and all carbapenems.

Class C β-lactamases, based on serine, are encoded by genes that are typically present in the bacterial chromosome. AmpC cephalosporinase is the main one, hydrolyzing penicillins and cephalosporins up to the third generation. AmpC includes different variants, referred to as PDCs (*Pseudomonas*-derived cephalosporinases). All examined isolates present different AmpC variants, with the *bla_PDC-3_* variant detected in three isolates and *bla_PDC-16_* in two of the isolates. The remaining variants were observed in only one strain.

The presence of the aminoglycoside class was confirmed in all isolates due to the presence of the *aph(3′)-IIb* gene. However, the gene aadA7 (also called *ant(3″)-Ia*) was exclusively detected in the HU141 strain. This gene confers resistance to gentamicin. The gene *ant(2″)-Ia* encodes an enzyme called nucleotidyltransferase, which modifies bacterial ribosomes, and it was detected in three of the isolates from Taiwan.

The genes *aac(6′)-Ib3* and *aac(6′)-Ib-cr* (a variant of the *aac(6′)-Ib* gene) are responsible for encoding an enzyme called aminoglycoside acetyltransferase, which modifies antibiotics, hindering their effective binding to bacterial targets; they were detected in isolates B19083-11 and 2910. In strain B21097-69, the gene *aac*(*6′*)-*IIa*, another variant of the *aac(6′)-Ib* gene, was also identified. The *aac(6′)-Ib-cr* gene can also confer resistance to quinolones, such as ciprofloxacin.

For antibiotics belonging to the fosfomycin and amphenicols classes, a significant prevalence was observed among bacterial isolates. The *fosA* and *fosX* genes, as examples of single-nucleotide phosphotransferase, were identified as conferring resistance to fosfomycin and fosfomycin tromethamine antibiotics, which are commonly used in the treatment of bacterial infections caused by *P. aeruginosa. fosA* was present in all isolates and *fosX* was identified in one isolate. The genes *catB2*, *catB7*, and *catB8* were detected among the bacterial isolates and correspond to different variants of the enzyme chloramphenicol acetyltransferase in *P. aeruginosa*. Remarkably, the *catB7* variant was the most commonly detected in the isolates. The *catB2* and *catB8* variants, on the other hand, were identified in only one of the isolates, which is relatively uncommon considering that the *catB8* gene is an additional variant of chloramphenicol acetyltransferase B found in *P. aeruginosa*.

The *sul1* gene associated with bacterial resistance to sulfonamide antibiotics was detected in five isolates.

The *tetG* gene in *P. aeruginosa* encodes an efflux pump protein that actively expels tetracycline antibiotics from the bacterial cell, reducing their intracellular concentration and effectiveness. This gene was identified in only one isolate.

### 3.4. Virulome

Genomic approaches to the virulome have unveiled the presence of numerous genes associated with virulence factors in *P. aeruginosa*, spanning aspects such as adherence, motility, antimicrobial activity, antiphagocytosis, secretion systems, iron uptake, and quorum sensing, among others. Concerning adherence, genes linked to flagellum production (e.g., *fliA-T*, *motA-D)*, the O-antigen of LPS, and type IV pili synthesis (e.g., *pilA* family genes, *fimT*, *fimU*, *fimV*) were identified, along with proteins related to type IV pili motility (e.g., *chpA*-*E*, *pilG* family genes).

Regarding antimicrobial activity, various virulence genes associated with phenazine biosynthesis, such as *phzA*, *phzA2*, *phzB1*, and *phzB2*, were detected.

In terms of antiphagocytosis, genes involved in alginate biosynthesis, such as *alg44*, *alg8*, *algA*, *algC*, *algD*, *algE* and others, were identified.

Biofilms play a crucial role in antimicrobial resistance, providing *P. aeruginosa* with structural and physiological protection that hinders the action of antibiotics and the immune system, making infections more persistent and difficult to eradicate. Several genes associated with biofilm formation were identified, including genes from the pel family (*pelA*, *pelB* and *pelC*), as well as the quorum sensing regulation system, mediated by *lasR*, *lasI*, *rhlR* and *rhlI*, responsible for biofilm maturation. Furthermore, different secretion systems were identified, notably the type VI secretion system (H-T6SS) and the type III secretion system (T3SS) of *P. aeruginosa.* Isolate 4098, belonging to ST4258, and isolate 2910, belonging to ST298, both contain the *exoU* gene. The *exoU* gene codes for the *exoU* toxin, a phospholipase-type enzyme that plays a crucial role in the pathogenicity of *P. aeruginosa*, facilitating cellular invasion and the destruction of host tissues.

### 3.5. Genomic Profiling

**Figure 2:**
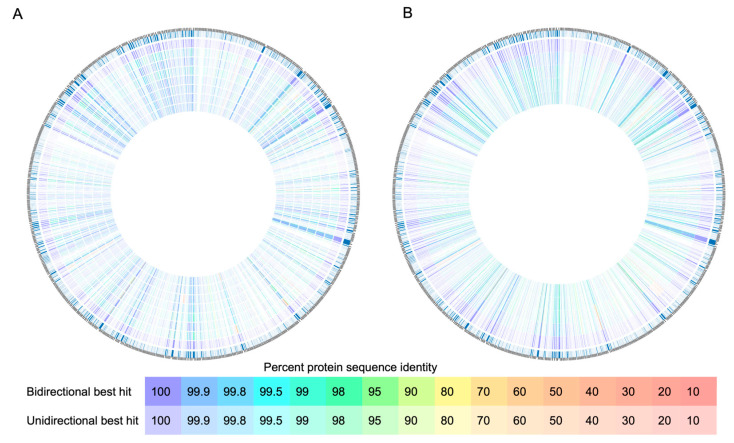
Graphical representation of the BLAST-based comparison of *P. aeruginosa* genomes using the BV-BRC tool. (**A**) Genome of the Portuguese isolates, where the outermost genome corresponds to *P. aeruginosa* PAO1, *P. aeruginosa* HW3, *P. aeruginosa* HSB35, *P. aeruginosa* HU141, *P. aeruginosa* HU134, *P. aeruginosa* HU130, *P. aeruginosa* HU122 and *P. aeruginosa* HU121. (**B**) Genome of the Taiwanese isolates, where the outermost genome corresponds to *P. aeruginosa* PAO1, *P. aeruginosa* B19083-11, *P. aeruginosa* B21097-69, *P. aeruginosa* 2910 and *P. aeruginosa* 4098.

The comparative analysis of colistin-resistant *P. aeruginosa* genomes revealed differences between samples from Portugal and Taiwan compared to the reference strain PAO1 (Figure 2).

The Taiwanese isolates exhibited divergences from the reference strain, while intra-strain differences were less pronounced. Deletions were observed between reference isolates and colistin-resistant isolates in various gene clusters, including genes crucial for heme utilization and adhesion, belonging to the ShlA/HecA/FhaA family (Supplementary Tables S1 and S2).

Clusters of genes associated with membrane proteins and hypothetical proteins were also affected by deletions. However, genes related to Lipid A and LPS alterations, such as *PmrA-PmrB*, *PhoP-PhoQ*, *ParR-ParS*, and the *ArnBCADTEF-PmrE* operon, remained highly conserved, with a high percentage of similarity (>95%).

The isolates of *P. aeruginosa* from Portugal exhibited significant divergences compared to both the reference isolates and among themselves, when compared to the isolates from Taiwan. Remarkably, complete deletions were observed in gene clusters associated with Chloramphenicol O-acetyltransferase (CatB Family), accessory cholera enterotoxin, and mRNA interferase RelE in isolates HU121 and HUW3, while these genes were present in other isolates, albeit with differences in genetic similarity.

Additionally, the Type IV fimbrial biogenesis protein PilV gene showed significant modifications in the Portuguese isolates compared to the reference strain, with isolates HW3 and HU141 displaying low genetic similarity, approximately between 30 and 50%.

Like the Taiwan isolates, deletions were observed in gene clusters associated with T1SS RTX-secreted agglutinin in the Portuguese isolates. However, genes related to colistin resistance remained highly conserved.

### 3.6. Analysis of Mutations in Colistin-Resistant P. aeruginosa

The various genomes were compared to identify point mutations and variations in genetic content, aiming to assess potential within-host diversity. Several non-functional SNPs were detected, along with deleterious protein alterations, complex mutations involving deletions and insertions, and SNPs with a functional impact on genes related to colistin resistance.

The analysis of genetic mutations in various bacterial isolates revealed a wide array of alterations associated with colistin-resistance in *P. aeruginosa* (Table 5).

The identified modifications in the *cprS* gene, which is part of the two-component regulatory system CprR-CprS, play a pivotal role in this process by regulating bacterial resistance systems, including those related to colistin. These mutations have the potential to induce variations in the expression of genes involved in antibiotic resistance, thus affecting the bacterium’s ability to withstand antimicrobial effects. See Supplementary Figure S2 for the alignment using Clustal Omega of the HW3 strain against gb|AAG06466.1|+|cprS, which is the reference strain in the Comprehensive Antibiotic Resistance Database.

Similarly, the mutation in the *pmrB* gene, belonging to the two-component regulatory system PmrA-PmrB, which results in the substitution of tryptophan by glycine at position 25 of the protein, has been associated with colistin resistance in *P. aeruginosa.* We detected mutations in the *phoQ* gene, part of the phoP-phoQ two-component regulatory system, across different bacterial isolates.

Indeed, it is intriguing that, despite strain B21097-69 having more mutations in genes directly related to colistin, it does not exhibit the highest MIC. To determine whether there is any correlation between the mutations and MIC, the mutational analysis took into account not only genes directly linked to colistin resistance but also those potentially associated with bacterial resistance reported in the literature, as well as efflux pumps. The strain 4098 has more nonsynonymous mutations in resistance-related genes compared to the other isolates, particularly in the *lepA* genes.

Upon examining the mutations within the *arnBCADTE*-*pmrE* operon, numerous non-synonymous mutations linked to these genes are apparent. The protein changes evidenced in the inner membrane L-Ara4N transferase ArnT were identified in all colistin-resistant clinical isolates (Table 6). These changes include the following amino acid substitutions: Val266Ile, Leu11del, Ala282Ser, Arg502Gln, Ile509Val, Ala267Ser, Ala330Val, Leu439Ile, Val88Phe, Thr443Ile, Arg446His, Cys7Trp, His151Tyr, Thr166Ile, Ala265Val, Asn346Asp, Leu493fs, Gly156Arg, Asp232Asn, and 16Arg535Leu.

The complex mutation CysSer312SerGly appears to be present in all isolates associated with the ArnA protein. For the genes *arnD*, *arnE*, and *arnF*, a limited number of mutations were observed, suggesting a relatively stable genetic profile in these regions. In arnD protein, mutations included Phe58Leu, Glu25Asp, and Ser272Asn, with a complex mutation involving Ala118Val and Val123Ala. The ArnE protein exhibited the following mutations: Ala109Val, Thr13Ser, and Arg28His. The ArnF protein mutations comprised Val14Met, with complex mutations involving Ala118Val and Ala55Thr, along with Val123Ala and Val113Met.

## 4. Discussion

*P. aeruginosa* is a widely distributed bacterium that thrives in various environments, including soil, water, and even hospital settings. It is prevalent in both natural and human-made habitats. However, its significance extends beyond just being a common bacterium. *P. aeruginosa* is particularly concerning in medical contexts due to its remarkable adaptability and its tendency to cause persistent infections, especially in individuals with weakened immune systems [24].

The results presented in this study provide a comprehensive overview of the genetic diversity and distribution of certain *P. aeruginosa* subtypes, along with their clinical implications.

Our phylogenetic analysis provides valuable insights into the evolutionary relationships among different isolates of *P. aeruginosa*. The phylogenetic tree reveals significant genetic diversity, with distinct clades and branches suggesting evolutionary divergences over time. The presence of bootstrap values at each division of the tree provides a measure of statistical confidence in these evolutionary relationships. It is interesting to note that there is no clear distinction between isolates from Portugal and Taiwan, pointing to a global spread of these *P. aeruginosa* subtypes and highlighting the importance of global surveillance and international collaboration in controlling bacterial infections. The arrangement of isolates in close or distant groups can be explained by multiple factors that influence both the evolution and the adaptation of these microorganisms to specific environments. For example, even though the two bloodstream isolates from Taiwan originated from the same institute, the divergence in their clustering suggests that they may have acquired distinct genetic profiles from different infectious sources. In many cases, bacteremia can originate from UTIs or lung infections, resulting in different genomic profiles, since each niche imposes specific selective pressures and favors the acquisition of certain virulence and resistance determinants [8,25]. Additionally, the phylogenetic proximity observed between one of the Taiwanese isolates, *P. aeruginosa* PAO1, and the Portuguese UTI isolates may indicate the existence of a common ancestor or even the occurrence of horizontal gene exchange, which is an important mechanism for the dissemination of resistance and virulence genes [26,27]. This hypothesis is reinforced by studies that indicate that isolates associated with urinary tract infections often share similar genetic profiles, even in geographically distant contexts, due to adaptation to hospital environments with the intensive use of antibiotics. Thus, the presented phylogenetic analysis not only highlights the global heterogeneity of *P. aeruginosa* but also showcases the complexity of the evolutionary and epidemiological processes that contribute to the formation of clusters. Even isolates originating from the same hospital environment can follow different evolutionary trajectories, which reinforces the importance of conducting in-depth epidemiological and molecular studies for the development of more effective control and treatment strategies [28]. Another study conducted a comparative genomic analysis of clinical isolates of *P. aeruginosa* isolated from eye and cystic fibrosis patients. The analysis revealed significant variation in the size of the accessory genome among the 22 isolates studied, which correlated with the presence of genomic islands, insertion sequences, and prophages. The isolates exhibited diversity in sequence type and were dissimilar to globally epidemic *P. aeruginosa* clones. Notably, a majority of the eye isolates from India clustered within a single lineage. Indian eye isolates possessed genes linked to resistance against various antibiotics, which were absent in Australian isolates regardless of the infection source [29]. Another study investigated the evolutionary genomics of *P. aeruginosa*’s adaptation to the CF lung environment, where it transitions from a free-living environmental strain to one causing chronic infection. Whole-genome sequencing of 1000 *P. aeruginosa* isolates, including those isolated from CF patients, revealed that CF isolates were distributed across the phylogeny, indicating no genetic predisposition for chronic infection within specific clades. However, isolates from the CF niche experienced stronger positive selection on core genes compared to those from environmental or acute infection sources, indicating recent adaptation to the lung environment [30]. These findings contribute valuable insights into the genomic diversity of *P. aeruginosa* across different infection types and geographical locations.

The presence of transposable elements in bacteria is a widely observed characteristic that is of great importance in terms of their biology and adaptation. Among these elements, Tn3, IS6, IS3, IS5 and IS21 stand out; they were identified in several bacterial lineages. These transposable elements play a fundamental role in the genomic plasticity of bacteria, allowing for horizontal gene transfer, chromosomal reorganization, and the acquisition of new phenotypic characteristics, such as antibiotic resistance and adaptation to different environments [31,32]. In Tn3, several IS elements were identified, including IS6, whose importance in generating clusters of clinically relevant antibiotic resistance genes is becoming increasingly evident, and whose members may utilize an unusual transposition pathway [33]. Although not exclusive to *P. aeruginosa*, IS26 is widely distributed in various bacteria and plays a significant role in the dissemination of antibiotic resistance and genome rearrangement, representing an important factor to consider in studies of bacterial resistance and genomic adaptation [33]. IS3 and IS5 elements were also detected in all isolates; they are recognized for possessing associated transposases that facilitate DNA mobility within the bacterial genome. These transposable elements play crucial roles in bacterial evolution, allowing for gene transfer between organisms and contributing to adaptation to different environments and conditions [34]. IS21 and IS6 elements are pivotal in shaping *P. aeruginosa*’s genome, enabling rapid adaptation to antibiotics and environmental stresses. IS21 and IS6 elements drive genome rearrangements and insertions, promoting the spread of resistance genes such as carbapenemases (e.g., OXA-2). These IS families are linked to globally disseminated *P. aeruginosa* clones (e.g., ST463), which combine multidrug resistance with virulence factors such as ExoU [35,36]. In *P. aeruginosa* CMC-097, IS6100 (an IS6-like element) was identified adjacent to the In2020 integron. It carries *tnpA2* and inverted repeats (IRs) that mediate recombination events. The IS6100 element (IAU57_09080) in CMC-097 facilitates the transfer of resistance genes within integrons, contributing to multidrug resistance [37,38]. In the clinical strain *P. aeruginosa* CMC-097, 13 copies of an IS21 family transposon were identified, contributing to genomic plasticity and resistance gene dissemination [39]. IS21 elements are frequently associated with carbapenem resistance integrons (e.g., In2020), which carry genes including *bla_OXA-2_* (β-lactamase) and *aacA27* (aminoglycoside acetyltransferase) [37,38,39,40].

The comparative analysis of resistance to various antipseudomonal agents reveals that the isolates exhibit resistance to multiple antibiotic classes, with a high prevalence of resistance to β-lactams, particularly class D enzymes. [40]. The *bla_OXA-396_* gene is the most common, followed by *bla_OXA-494_*. Other genes such as *bla_OXA-846_*, *bla_OXA-488_*, *bla_OXA-848_*, *bla_OXA-395_*, and *bla_OXA-903_* were detected in two of the examined isolates [41]. The class B carbapenemases genes, including *bla_VIM-3_*, were found in isolates 4098 and 2910, which contribute to resistance to a broad range of antibiotics. The detection of MBL, such as *bla_VIM-3_*, is particularly concerning due to its ability to confer resistance to a broad range of antibiotics, complicating the treatment of bacterial infections [42,43,44]. The isolate B19083-11 is the only one to contain the *bla_SHV-12_* gene. Unlike other classes, class B has a metal-based structure, referred to as MBL.

The AmpC cephalosporinase, mainly represented by the *bla_PDC-3_* and *bla_PDC-16_* variants, was observed in all isolates. Increased expression of AmpC is associated with β-lactam resistance, particularly for combinations like ceftolozane/tazobactam and ceftazidime/avibactam [45,46,47,48]. Many different types of PDC have been documented, with certain variants associated with heightened resistance to ceftolozane/tazobactam and ceftazidime/avibactam [47,48,49,50]. Among isolates in the CC274 collection, a mutator strain named AUS601 displays significant resistance to ceftazidime, cefepime, and aztreonam, even without an increase in *AmpC* expression [51].

Additionally, various efflux pump genes, such as MexAB-OprM, MexCD-OprJ, and MexEF-OprN, were identified. These pumps contribute to multidrug resistance, complementing other mechanisms such as β-lactamase production and altered membrane permeability [52]. A mutational analysis of various genes revealed that the diverse isolates harbored mutations, primarily synonymous mutations in porins, multidrug efflux pumps, and membrane proteins, which are the primary drivers of prevalent carbapenem resistance [53,54,55]. During chronic CF respiratory infections, the MexAB-OprM efflux pump is subjected to significant mutational pressure, including the occurrence of inactivating mutations. This observation aligns with previous studies indicating that this efflux system is not essential and thus may be lost or deactivated in favor of overexpressing MexXY-OprM in certain subpopulations of CF *P. aeruginosa* [56].

The genes *aac(6′)-Ib3*, *aac(6′)-Ib-cr*, *and aac(6′)-IIa*, found in isolates B19083-11, 2910, and B21097-69, respectively, may confer resistance to aminoglycosides, while the gene *aac(6′)-Ib-Hangzhou*, identified in strain 2910 from Taiwan, is associated with *Enterobacteria* and also confers resistance to aminoglycosides [57,58,59] Additionally, the gene *fosA*, present in all isolates, and the gene *fosX*, identified in one isolate, confer resistance to antibiotics belonging to the classes of fosfomycin [60]. *sul1* was detected in five isolates. Sul1 proteins confer resistance, which can be a significant public health concern [61,62].

The comparison between Portugal and Taiwan regarding antibiotic use and antibiotic stewardship strategies allows us to contextualize the resistance profiles observed in this study. In Portugal, antibiotic consumption has historically been high, especially in the community sector [63], although recent measures, such as the Prevention and Control of Antimicrobial Resistance Program (PPCRA), have been implemented to reduce inappropriate use [64]. Data from the European Centre for Disease Prevention and Control (ECDC) indicate a downward trend in total antibiotic consumption in recent years, in line with European guidelines for the rational use of antimicrobials [65].

On the other hand, Taiwan has a robust system for monitoring antibiotic use, with strict regulations established by the Taiwan National Antimicrobial Resistance Action Plan (TNARAP), which includes the strict control of prescriptions and the promotion of antibiotic stewardship programs in hospitals [66,67]. A study conducted by Lee et al. (2012) showed that, despite the high consumption of antibiotics in Taiwan, government initiatives have been effective in reducing unnecessary use, especially in hospital settings [66].

When comparing data from both countries, it is observed that, although Portugal and Taiwan face similar challenges in combating antimicrobial resistance, Taiwan implements a more centralized and rigorous control of prescriptions, while Portugal has focused on raising awareness and educating health professionals. The relationship between these different management styles and the resistance profiles identified in this study may provide important insights into the impact of national policies on the development of resistance in *P. aeruginosa*.

The comparative analysis of colistin-resistant *P. aeruginosa* genomes highlights divergences between isolates from Portugal and Taiwan when compared to the reference strain PAO1. Taiwanese isolates showed variations from the reference, with fewer intra-strain differences. Deletions were noted in various gene clusters, including those crucial for heme utilization and adhesion (ShlA/HecA/FhaA family). Particularly noteworthy is a large exoprotein whose function is presumably linked to heme utilization or adhesion, suggesting its importance for the virulence and persistence of *P. aeruginosa*, especially in nosocomial environments. Such deletions may lead to reduced virulence, colonization capacity, and altered genetic mobility, affecting bacterial evolution and antibiotic resistance acquisition [67]. *P. aeruginosa* biofilms represent a significant barrier to the efficacy of antibiotics, contributing to the chronicity and resistance of infections. Several genes are involved in biofilm formation and maintenance, including *pelA*, *pelB* and *pelC*, which encode to produce extracellular matrix polysaccharides, and *pslA*, *pslB* and *pslC*, which are essential for cell adhesion and biofilm structure [68]. Furthermore, the *algD* gene is directly related to the production of alginate, promoting resistance to the immune system and antibiotics [69]. The quorum-sensing regulatory system, mediated by *lasR*, *lasI*, *rhlR* and *rhlI*, is another key factor in biofilm maturation and the expression of virulence factors. The presence of biofilms not only protects bacteria against antimicrobial agents, but also favors the spread of resistance genes, worsening the challenges in treating infections caused by *P. aeruginosa* [70].

Clusters of genes related to T1SS RTX-secreted agglutinins underwent deletions, potentially reducing adhesion and toxin secretion, as well as altering the host immune response. Despite these deletions, colistin resistance genes remained conserved, indicating their importance [71,72].

Portuguese isolates exhibited significant genetic divergence, including complete deletions in some gene clusters and modifications in others, such as the PilV gene. These changes likely result from natural selection in response to environmental pressures, such as colistin exposure in hospitals. Molecular evolution also plays a role in the observed genetic variation, reflecting the bacteria’s adaptation to different environments.

The analysis of mutations in colistin-resistant *P. aeruginosa* identified various alterations, including non-functional SNPs, deleterious protein alterations, complex mutations involving deletions and insertions, and SNPs impacting genes related to colistin resistance. Mutations in regulatory networks, such as those in the *cprS*, *pmrB*, *phoQ*, *parS*, and *parR* genes, were identified, potentially affecting antibiotic resistance mechanisms. Alterations in these regulatory elements can modulate the expression of genes involved in colistin resistance pathways, thereby influencing the overall resistance phenotype of the bacterium [73]. Furthermore, the discrepancy between the mutation profile of the B21097-69 strain and its MIC underscores the complexity of the relationship between genotype and phenotype in antibiotic resistance. While this strain harbors more mutations directly related to colistin, its MIC does not necessarily reflect the highest resistance level observed among the isolates analyzed. This discrepancy suggests that factors beyond the presence of specific mutations may contribute to the overall resistance phenotype, such as the genetic background, epistatic interactions, or additional resistance mechanisms. Additionally, strain 4098 exhibited more nonsynonymous mutations in resistance-related genes, indicating potential implications for antibiotic resistance mechanisms. These mutations may alter the function or expression of key resistance determinants, leading to variations in resistance levels among bacterial isolates. Interpreting the mechanisms underlying colistin resistance presents challenges due to the intricate nature of bacterial response systems. Mutations in two-component regulators do not consistently correlate with clinical colistin resistance, indicating a potential interplay between various regulatory pathways. For instance, some studies suggest that individual two-component systems may not singularly dictate colistin resistance acquisition in *P. aeruginosa*, hinting at complex interactions between regulatory elements [74,75]. The kinase sensor PmrB activates the transcriptional response regulator PmrA either through a phosphotransfer relay or because of a mutation in *pmrB*. This two-component system drives bacterial responses to multiple stimuli and regulates modifications of the LPS [76,77]. In *P. aeruginosa* isolates B21097-69, 2910, and 4098, mutations in the *pmrB* gene have been identified. Specifically, strain 2910 exhibits two types of mutations (Trp25Gly and Gly469fs). The Ala247thr mutation detected in isolate B21097-69 involves lipid A and is generally associated with gain of function [78,79].

The detection of mutations in the *phoQ* gene across various bacterial isolates indicates that this gene may play a role in antibiotic resistance, including resistance to colistin. One specific mutation identified is both a frameshift and missense mutation, causing a shift in the reading frame and leading to a missense change at codon position 152. This alteration may have substantial implications for the structure and function of the PhoQ protein, which is essential to regulating bacterial resistance systems [80]. Yang et al. [75] showed that individual two-component systems may not be essential for the acquisition of colistin resistance in *P. aeruginosa*. However, it should be noted that the isolate with a premature stop codon in *phoQ* exhibited a high level of resistance [75]. The mutation in the *phoQ* gene described as a frameshift and missense (Val152fs) significantly impacts the function of the PhoQ protein. A frameshift mutation, such as Val152fs, causes a shift in the reading frame of the gene, leading to an altered and often truncated protein product. This specific mutation can result in a nonfunctional or partially functional PhoQ protein, disrupting its ability to respond to environmental signals and regulate target genes effectively. In the context of colistin resistance, mutations in phoQ can lead to either the increased or decreased expression of genes that modify LPS on the bacterial surface. These modifications, such as the addition of L-Ara4N or phosphoethanolamine to lipid A, reduce the negative charge of LPS and decrease the colistin binding affinity [76,77]. In *P. aeruginosa*, mutations in the *phoQ* gene, along with mutations in other regulatory genes such as *pmrB*, can enhance resistance to colistin by upregulating the *arnBCADTEF* operon, which encodes enzymes for LPS modification. The resulting changes in the outer membrane structure help the bacteria evade the antimicrobial activity of colistin, thus contributing to high levels of resistance [77].

Colistin resistance in *P. aeruginosa* can also be associated with alterations in LPS, particularly in lipid A, involving the operons *pmrAB* and *arnBCADTEF-pmrE*, which mediate the synthesis and transfer of pEtN and L-Ara4N, respectively [4]. The *arnA* gene mutation (*arnA* complex mutation; CysSerProGln312SerGlyProLys) introduces a complex amino acid change at position 312. ArnA is crucial in the biosynthesis of UDP- L-Ara4N, a key molecule in the lipid A modification of LPS [77,78,79,80]. These extensive changes can significantly alter the function of ArnA, impacting the overall resistance mechanism. The SNPs in the *arnT* gene cause multiple amino acid substitutions. ArnT is responsible for transferring L-Ara4N to lipid A. Each mutation can individually or collectively affect the enzyme’s efficiency, altering the bacterial membrane’s structure and enhancing resistance to colistin. The mutations in these genes collectively contribute to modifying the outer membrane of *P. aeruginosa*, reducing colistin’s ability to bind and exert its antimicrobial effect [78,79,80]. However, the phenotype of colistin resistance typically arises from a complex interplay of multiple mechanisms that enable the bacteria to evade antibiotic stress. In addition to modifications in LPS, *P. aeruginosa* can develop resistance through the overexpression of efflux pumps, which expel the antibiotic from the cell, and the production of enzymes that inactivate colistin before it can exert its bactericidal effect [53,80]. These mechanisms often work together, creating a robust defense system that makes treating *P. aeruginosa* infections particularly challenging.

## 5. Conclusions

*P. aeruginosa* is a significant pathogen that is responsible for serious hospital-acquired infections, particularly in immunocompromised patients and those with cystic fibrosis. It is one of the primary organisms responsible for nosocomial infections, including pneumonia and UTIs.

The genetic diversity and distribution of its specific subtypes show the pathogen’s adaptability to various environments, with UTIs being the most common source. High-risk clones such as ST235, associated with widespread dissemination and high antibiotic resistance, pose a significant public health threat. The phylogenetic analysis revealed no clear distinction between isolates from Portugal and Taiwan, highlighting its global spread. Mutations in genes such as *phoQ* and *pmrB*, and in the *arnBCADTEF* operon, contribute to colistin resistance by altering the LPS structure. This study aimed to provide genomic data from isolates from two countries, which are useful for identifying the determinants of antibiotic resistance, as well as prospecting data for potential new therapeutic targets.

## Figures and Tables

**Table 1:** Sample of colistin-resistant *P. aeruginosa* isolates.

Isolate Reference No.	Biosample Accession	Host Disease	Isolation Source	Year	Geolocation
HU121	SAMN40216622	Urinary tract infection	Urine	2022	Portugal
HU122	SAMN40216623	Urinary tract infection	Urine	2022	Portugal
HU130	SAMN40216624	Urinary tract infection	Urine	2022	Portugal
HU134	SAMN40216625	Urinary tract infection	Urine	2022	Portugal
HU141	SAMN40216632	Urinary tract infection	Urine	2022	Portugal
HW3	SAMN40216626	Wound infection	Skin wound	2022	Portugal
HSB35	SAMN40216631	Pulmonary infection	Bronchial secretion	2022	Portugal
4098	SAMN40216628	Urinary tract infection	Urine	2012	Taiwan
2910	SAMN40216627	Urinary tract infection	Urine	2010	Taiwan
B19083-11	SAMN40216629	Bacteremia	Blood	2019	Taiwan
B21097-69	SAMN40216630	Bacteremia	Blood	2021	Taiwan

**Table 2:** Genomic information for the isolates (*), encompassing genome size (in base pairs), DNA GC percentage, contigs, protein-coding genes, rRNA genes, and RNA genes.

*P. aeruginosa* Reference No.	Genome Size (bp)	DNA GC (%)	Protein Coding Genes	rRNA Genes	tRNA Genes	ST	Serotype
HU121	6,474,782	66.39	6133	5	58	2438	O6
HU122	6,488,087	66.36	6163	5	58	270	O2
HU130	6,707,783	66.04	6450	7	60	348	O12
HU134	6,361,249	66.48	5997	6	56	4258	O6
HU141	6,983,523	65.85	6752	4	63	253	O12
HW3	6,774,228	65.97	6486	7	59	1601	O11
HSB35	6,307,730	66.48	5966	3	62	1053	O2
4098	7,003,552	65.95	6880	12	63	4258	O11
2910	6,962,389	66.00	6708	12	65	298	O11
B19083-11	6,713,745	66.10	6472	11	63	3002	O5
B21097-69	7,017,151	66.06	6977	12	64	235	O11

(*) Acquired using the BV-BRC v3.32.31a tool; sequence types (ST) were obtained using the MLST v2.0.9 tool, while serotypes were identified using the PAst v1.0 tool.

**Table 3:** Antimicrobial resistance phenotypes of *P. aeruginosa.*

Class and⁄or Antimicrobial	Breakpoints (mm; S≥/R<)	MIC (mg/L; S≥/R<)	Number of Resistant Isolates
Ampicillin	-	-	1
Amoxicillin/Clavulanate	-	-	2
Piperacillin	50/18	0.001/16	9
Piperacillin/Tazobactam	50/18	0.001/16	11
Ceftazidime	50/17	0.001/8	10
Cefepime	50/21	0.001/8	11
Doripenem	50/22	0.001/2	11
Ertapenem	-	-	2
Imipenem	50/20	0.001/4	9
Meropenem	20/14	02/8	6
Aztreonam	50/18	0.001/16	4
Ciprofloxacin	50/26	0.001/0.5	11
Levofloxacin	50/18	0.001/2	8
Amikacin	15/15	16/16	3
Tobramicyn	18/18	02/f2	1
Gentamicin	15/15	-	5
Colistin *	-	2 *	11

* The decision to utilize a breakpoint of 2 μg/mL for interpreting the colistin susceptibility results, despite the EUCAST recommendation of 4 μg/mL, is informed by recent discussions surrounding the efficacy and limitations of colistin. Concerns regarding inadequate drug exposure in patients with normal renal function, the inability to achieve bacterial stasis in pneumonia despite adequate exposure, and increased mortality rates associated with colistin monotherapy have prompted a reevaluation of breakpoints. The introduction of brackets in the proposed breakpoint tables (v12.0) aims to address these complexities, ensuring that communication reflects the nuances of colistin susceptibility and the need to combine therapy in systemic infections. Therefore, the decision to use a breakpoint of 2 μg/mL aligns with the evolving understanding of colistin’s role in therapy and the imperative to consider combination therapy in systemic infections, as outlined in the proposed EUCAST guidelines [23].

**Table 4:** Characteristics and distribution of the genotypes detected among the examined *P. aeruginosa* isolates.

Isolates	Phenotype Resistance	Resistance Genes
HU121	PIP/TAZ CAZ CIP FEP DOR IMP LVX PIP	*aph(3′)-Iib*, *catB7*, *bla_PDC-3_*, *bla_OXA-486_*, *bla_OXA-903_*, *fosA*
HU122	ERT PIP/TAZ CAZ CIP IMP LVX FEP DOR PIP	*aph(3′)-Iib*, *catB7*, *bla_PDC-8_*, *bla_OXA-486_*, *bla_OXA-903_*, *fosA*
HU130	PIP/TAZ CAZ CIP FEP DOR IMP MEM LVX PIP	*aph(3′)-Iib*, *catB8*, *bla_PDC-5_*, *bla_OXA-396_*, *bla_OXA-494_*, *fosA*, *sul1*
HU134	ERT PIP/TAZ CIP FEP DOR IMP PIP	*aph(3′)-Iib*, *catB7*, *bla_PDC-3_*, *bla_OXA-396_*, *bla_OXA-494_*, *fosA*
HU141	PIP/TAZ CAZ CIP FEP DOR GM TOB IMP LVX MEM PIP	*aph(3′)-Iib*, *aadA7*, *catB7*, *bla_PDC-34_*, *bla_OXA-488_*, *fosA*, *sul1*
HW3	AMP ATM CAZ CIP FEP DOR IMP LVX MEM PIP TZP	*aph(3′)-Iib*, *catB7*, *bla_PDC-39_*, *bla_OXA-396_*, *bla_OXA-494_*, *fosA*,
HSB35	ATM CAZ CIP FEP DOR LVX PIP TZP	*aph(3′)-Iib*, *catB7*, *bla_OXA-396_*, *bla_OXA-847_*, *bla_OXA-494_*, *bla_PCD-1_*, *fosA*, *crpP*
4098	AK AMC ATM CAZ CIP FEP DOR GM IPM MEM PIP TZP	*aph(3′)-Iib*, *catB7*, *bla_VIM-3_*, *bla_PDC-16_*, *bla_OXA-395_*, *bla_OXA-848_*, *fosA*, *sul1*, *qacE*
2910	AK ATM CAZ CIP FEP DOR GM IPM MEM PIP TZP	*aph(3′)-IIb*, *aac(6′)-Ib3*, *aac(6′)-Ib-cr*, *aac(6′)-Ib-Hangzhou*, *ant(2″)-Ia*, *catB7*, *bla_VIM-3_*, *bla_PDC-16_*, *bla_OXA-395_*, *bla_OXA-848_*, *fosA*, *fosX*, *sul1*, *crpP*, *qacE*
B19083-11	AK AMC CAZ CIP FEP DOR GM IPM LVX MEM TZP	*aph(3′)-IIb*, *aac(6′)-Ib3*, *aac(6′)-Ib-cr*, *ant(2″)-Ia*, *catB7*, *bla_PDC-3_*, *bla_OXA-10_*, *bla_OXA-50_*, *bla_SHV-12_*, *fosA*, *sul1*, *crpP*
B21097-69	CAZ CIP FEP DOR GM LVX TZP	*aph(3′)-IIb*, *aac(6′)-IIa*, *ant(2″)-Ia*, *catB2*, *catB7*, *bla_PDC-35_*, *bla_OXA-488_*, *bla_OXA-17_*, *fosA*, *crpP*, *tet(G)*, *floR*, *qacE*

pip (piperacillin); pip/taz (piperacillin/tazobactam); caz (ceftazidime); fep (cefepime); dor (doripenem); ipm (imipenem); mem (meropenem); ert (ertapenem); atm (aztreonam); amp (ampicillin); amc (amoxicillin/clavulanate); cip (ciprofloxacin); lvx (levofloxacin); gm (gentamicin); tob (tobramycin); ak (amikacin).

**Table 5:** Non-synonymous variations in regulatory networks and specific loci reported to have possible associations with colistin resistance in *P. aeruginosa.*

Isolates	Related Genes	Type of Mutation	Gene and Protein Change	Locus_Tag
HU134	*cprS*	snp	missense_variant c.347C > T p.Ala116Val	PA3078
	*parS*	snp	missense_variant c.1033G > A p.Ala345Thr	PA1798
HU141	*parS*	snp	missense_variant c.467C > T p.Pro156Leu	PA1798
HW3	*cprS*	snp	missense_variant c.322G > A p.Asp108Asn	PA3078
			missense_variant c.475G > A p.Val159Ile	
			missense_variant c.1158G > C p.Glu386Asp	
2910	*parR*	snp	missense_variant c.695G > A p.Gly232Asp	PA1799
	*pmrB*	snp	missense_variant c.73T > G p.Trp25Gly	PA4777
		ins	frameshift_variant c.1403dupG p.Gly469fs	
	*rsmA*	del	non_coding_transcript_variant	PA0905
4098	*parR*	snp	missense_variant c.695G > A p.Gly232Asp	PA1799
	*pmrB*	snp	missense_variant c.739G > A p.Ala247Thr	PA4777
	*rsmA*	del	non_coding_transcript_variant	PA0905
B19083-11	*cprS*	snp	missense_variant c.1282G > T p.Ala428Ser	PA3078
	*parR*	snp	missense_variant c.259G > A p.Glu87Lys	PA1799
	*phoQ*	complex	frameshift_variant&missense_variant c p.Val152fs	PA1180
	*rsmA*	del	non_coding_transcript_variant	PA0905
B21097-69	*cprS*	snp	missense_variant c.47C > G p.Thr16Ser	PA3078
		ins	frameshift_variant c.209_210insT p.Phe71fs	
		del	disruptive_inframe_deletion c.273_275delGCC p.Pro92del	
		snp	missense_variant c.1158G > C p.Glu386Asp	
	*parS*	ins	frameshift_variant c.248_249insA p.Gln84fs	PA1798
	*parR*	snp	missense_variant c.509G > A p.Ser170Asn	PA1799
		snp	synonymous_variant c.465C > A p.Ile155Ile	
	*pmrB*	snp	missense_variant c.739G > A p.Ala247Thr	PA4777
	*phoQ*	complex	missense_variant c.254_258delACGACinsTCGAT p.Tyr85Phe	PA1180
	*rsmA*	ins	non_coding_transcript_variant	PA0905
		del	non_coding_transcript_variant	

snp—single-nucleotide polymorphism; ins—insertion; del—deletion; complex—more intricate variations or mutations in the DNA sequence.

**Table 6:** Non-synonymous variations of the *arnBCADTE* operon associated with colistin resistance in *P. aeruginosa.*

Isolates	Related Genes	Type of Mutation	Protein Change	Locus_Tag
HU121	*arnB*	snp	Val302Ala	PA3552
	*arnA*	complex	CysSer312SerGly	PA3554
	*arnT*	snp	Val266Ile	PA3556
HU122	*arnB*	snp	Val302Ala	PA3552
	*arnC*	snp	Arg198His	PA3553
	*arnA*	complex	CysSer312SerGly	PA3554
	*arnT*	del	Leu11del	PA3556
		snp	Val266Ile	
		snp	Ala282Ser	
		snp	Arg502Gln	
		snp	Ile509Val	
HU130	*arnB*	snp	Val302Ala	PA3552
	*arnC*	del	Ala274_Phe277del	PA3553
	*arnA*	complex	CysSer312SerGly	PA3554
	*arnT*	snp	Ala267Ser	PA3556
		snp	Ala330Val	
		snp	Leu439Ile	
		snp	Arg502Gln	
		snp	lle509Val	
	*arnF*	snp	Val14Met	PA3558
HU134	*arnB*	snp	Lys286Glu	PA3552
		snp	Val302Ala	
	*arnA*	complex	CysSer312SerGly	PA3554
		snp	Ile388Val	
	*arnD*	snp	Phe58Leu	PA3555
	*arnT*	snp	Val88Phe	PA3556
		snp	Thr443Ile	
		snp	Arg446His	
		snp	Arg502Gln	
		snp	Ile509Val	
	*arnF*	snp	Val14Met	PA3558
HU141	*arnB*	snp	Val302Ala	PA3552
		snp	Glu376Asp	
	*arnC*	snp	Ala327Val	PA3553
	*arnA*	complex	CysSer312SerGly	PA3554
		snp	Ile388Val	
	*arnD*	snp	Glu25Asp	PA3555
		complex	Ala118Val	
		snp	Val123Ala	
	*arnT*	snp	Cys7Trp	PA3556
		complex	His151Tyr	
		snp	Thr166Ile	
		snp	Ala265Val	
		snp	Ile509Val	
	*arnE*	snp	Ala109Val	PA3557
	*arnF*	snp	Val14Met	PA3558
		complex	Ala55Thr	
HW3	*arnB*	snp	Val302Ala	PA3552
		snp	Glu376Asp	
	*arnA*	complex	Phe80Tyr	PA3554
		snp	Thr297Ala	
		complex	CysSer312SerGly	
		snp	Ile388Val	
	*arnD*	snp	Phe58Leu	PA3555
	*arnT*	snp	His151Tyr	PA3556
		snp	Ala267Ser	
		snp	Leu337Gln	
		snp	Thr443Ala	
		snp	Ile509Val	
	*arnF*	snp	Val113Met	PA3558
HSB35	*arnB*	snp	Lys286Glu	PA3552
		snp	Val302Ala	
	*arnA*	complex	CysSer312SerGly	PA3554
		snp	Gln661Leu	
	*arnD*	snp	Ser272Asn	PA3555
	*arnT*	snp	Cys7Trp	PA3556
		snp	Ala267Ser	
		snp	Arg446His	
		snp	Val511Met	
2910	*arnB*	snp	Thr143Ala	PA3552
		snp	Lys286Glu	
		snp	Val302Ala	
		snp	Arg340Cys	
		snp	Glu375Lys	
		snp	Glu376Asp	
	*arnC*	snp	Ala327Val	PA3553
	*arnA*	complex	CysSer312SerGly	PA3554
		snp	Ile388Val	
	*arnD*	snp	Glu25Asp	PA3555
		complex	Ala118Val	
		snp	Val123Ala	
	*arnT*	complex	His151Tyr	PA3556
		snp	Thr166Ile	
		snp	Ala265Val	
		snp	Thr443Ala	
		snp	Ile509Val	
	*arnE*	snp	Thr13Ser	PA3557
4098	*arnB*	snp	Thr143Ala	PA3552
		snp	Lys286Glu	
		snp	Val302Ala	
		snp	Arg340Cys	
		snp	Glu375Lys	
		snp	Glu376Asp	
	*arnC*	snp	Ala327Val	PA3553
	*arnA*	complex	CysSer312SerGly	PA3554
		snp	Ile388Val	
	*arnD*	snp	Glu25Asp	PA3555
		complex	Ala118Val	
		snp	Val123Ala	
	*arnT*	complex	His151Tyr	PA3556
		snp	Thr166Ile	
		snp	Ala265Val	
		snp	Asn346Asp	
		snp	Thr443Ala	
		complex	Leu493fs	
		snp	Ile509Val	
	*arnE*	snp	Thr13Ser	PA3557
B19083-11	*arnB*	snp	Val302Ala	PA3552
	*arnA*	complex	CysSerProGln312SerGlyProLys	PA3554
	*arnT*	snp	Gly156Arg	PA3556
		snp	Asp232Asn	
		snp	Ala267Ser	
		snp	Arg446His	
		snp	Ile509Val	
B21097-69	*arnB*	snp	Arg105Ser	PA3552
		snp	Val302Ala	
		snp	Glu376Asp	
	*arnC*	snp	Ala327Val	PA3553
	*arnA*	complex	CysSer312SerGly	PA3554
		snp	Ile388Val	
		ins	Ala662fs	
	*arnD*	snp	Phe58Leu	PA3555
		snp	Ser272Asn	
	*arnT*	snp	Cys7Trp	PA3556
		complex	His151Tyr	
		snp	Thr166Ile	
		snp	Ala265Val	
		snp	Thr443Ala	
		snp	Val468Met	
		snp	Ile509Val	
		snp	16Arg535Leu	
	*arnE*	snp	Arg28His	PA3557
		snp	Ala109Val	
	*arnF*	snp	Val14Met	PA3558

## Data Availability

Data are contained within the article and Supplementary Materials.

## Supplementary Materials

The following supporting information can be downloaded at: https://www.mdpi.com/article/10.3390/pathogens14040387/s1, Figure S1: Presence of IS26 in P. aeruginosa strain B19083-1; Figure S2: Alignment of Pseudomonas aeruginosa HW3 Strain with the Reference Strain cprS (gb|AAG06466.1|) using Clustal Omega; Table S1: Genomic annotating using BV-BRC tool for Portuguese isolates; Table S2:Genomic annotating using BV-BRC tool for Tawain isolates.
