# Supplementary material for: Mutational Analysis of Colistin-Resistant Pseudomonas aeruginosa Isolates: From Genomic Background to Antibiotic Resistance
Source: Pathogens. 2025 Apr 15;14(4):387. doi: 10.3390/pathogens14040387 (PMC12030098; doi:10.3390/pathogens14040387)
Supplement: Supplementary file 1 [file pathogens-14-00387-s001.zip › Supplementary Figure.pdf]

```
> IS26  Family: IS6
Length=820

 Score = 1620 bits (817),  Expect = 0.0
 Identities = 817/817 (100%), Gaps = 0/817 (0%)
 Strand=Plus/Minus

Query  1    ACTGTTGCAAAGTTAGCGATGAGGCAGCCTTTTGTCTTATTCAAAGGCCTTACATTTCAA  60
            ||||||||||||||||||||||||||||||||||||||||||||||||||||||||||||
Sbjct  817  ACTGTTGCAAAGTTAGCGATGAGGCAGCCTTTTGTCTTATTCAAAGGCCTTACATTTCAA  758

Query  61   AAACTCTGCTTACCAGGCGCATTTCGCCCAGGGGATCACCATAATAAAATGCTGAGGCCT  120
            ||||||||||||||||||||||||||||||||||||||||||||||||||||||||||||
Sbjct  757  AAACTCTGCTTACCAGGCGCATTTCGCCCAGGGGATCACCATAATAAAATGCTGAGGCCT  698

Query  121  GGCCTTTGCGTAGTGCACGCATCACCTCAATACCTTTGATGGTGGCGTAAGCCGTCTTCA  180
            ||||||||||||||||||||||||||||||||||||||||||||||||||||||||||||
Sbjct  697  GGCCTTTGCGTAGTGCACGCATCACCTCAATACCTTTGATGGTGGCGTAAGCCGTCTTCA  638

Query  181  TGGATTTAAATCCCAGCGTGGCGCCGATTATCCGTTTCAGTTTGCCATGATCGCATTCAA  240
            ||||||||||||||||||||||||||||||||||||||||||||||||||||||||||||
Sbjct  637  TGGATTTAAATCCCAGCGTGGCGCCGATTATCCGTTTCAGTTTGCCATGATCGCATTCAA  578

Query  241  TCACGTTGTTCCGGTACTTAATCTGTCGGTGTTCAACGTCAGACGGGCACCGGCCTTCGC  300
            ||||||||||||||||||||||||||||||||||||||||||||||||||||||||||||
Sbjct  577  TCACGTTGTTCCGGTACTTAATCTGTCGGTGTTCAACGTCAGACGGGCACCGGCCTTCGC  518

Query  301  GTTTGAGCAGAGCAAGCGCGCGACCATAGGCGGGCGCTTTATCCGTGTTGATGAATCGCG  360
            ||||||||||||||||||||||||||||||||||||||||||||||||||||||||||||
Sbjct  517  GTTTGAGCAGAGCAAGCGCGCGACCATAGGCGGGCGCTTTATCCGTGTTGATGAATCGCG  458

Query  361  GGATCTGCCACTTCTTCACGTTGTTGAGGATTTTACCCAGAAACCGGTATGCAGCTTTGC  420
            ||||||||||||||||||||||||||||||||||||||||||||||||||||||||||||
Sbjct  457  GGATCTGCCACTTCTTCACGTTGTTGAGGATTTTACCCAGAAACCGGTATGCAGCTTTGC  398

Query  421  TGTTACGACGGGAGGAGAGATAAAAATCGACAGTGCGGCCCCGGCTGTCGACGGCCCGGT  480
            ||||||||||||||||||||||||||||||||||||||||||||||||||||||||||||
Sbjct  397  TGTTACGACGGGAGGAGAGATAAAAATCGACAGTGCGGCCCCGGCTGTCGACGGCCCGGT  338

Query  481  ACAGATACGCCCAGCGGCCATTGACCTTCACGTAGGTTTCATCCATGTGCCACGGGCAAA  540
            ||||||||||||||||||||||||||||||||||||||||||||||||||||||||||||
Sbjct  337  ACAGATACGCCCAGCGGCCATTGACCTTCACGTAGGTTTCATCCATGTGCCACGGGCAAA  278

Query  541  GATCGGAAGGGTTACGCCAGTACCAGCGCAGCCGTTTTTCCATTTCAGGCGCATAACGCT  600
            ||||||||||||||||||||||||||||||||||||||||||||||||||||||||||||
Sbjct  277  GATCGGAAGGGTTACGCCAGTACCAGCGCAGCCGTTTTTCCATTTCAGGCGCATAACGCT  218

Query  601  GAACCCAGCGGTAAATCGTGGAGTGATCGACATTCACTCCGCGTTCAGCCAGCATCTCCT  660
            ||||||||||||||||||||||||||||||||||||||||||||||||||||||||||||
Sbjct  217  GAACCCAGCGGTAAATCGTGGAGTGATCGACATTCACTCCGCGTTCAGCCAGCATCTCCT  158

Query  661  GCAGCTCACGGTAACTGATGCCGTATTTGCAGTACCAGCGTACGGCCCACAGAATGATGT  720
            ||||||||||||||||||||||||||||||||||||||||||||||||||||||||||||
Sbjct  157  GCAGCTCACGGTAACTGATGCCGTATTTGCAGTACCAGCGTACGGCCCACAGAATGATGT  98

Query  721  CACGCTGAAAATGCCGGCCTTTGAATGGGTTCATGTGCAGCTCCATCAGCAAAAGGGGAT  780
            ||||||||||||||||||||||||||||||||||||||||||||||||||||||||||||
Sbjct  97   CACGCTGAAAATGCCGGCCTTTGAATGGGTTCATGTGCAGCTCCATCAGCAAAAGGGGAT  38

Query  781  GATAAGTTTATCACCACCGACTATTTGCAACAGTGCC  817
            |||||||||||||||||||||||||||||||||||||
Sbjct  37   GATAAGTTTATCACCACCGACTATTTGCAACAGTGCC  1
```

Supplementary Figure S1. Presence of IS26 in P. aeruginosa strain B19083-1. While IS26 is not exclusive to *P. aeruginosa*, its detection in this strain highlights its widespread distribution across various bacterial species. S26 plays a crucial role in the dissemination of antibiotic resistance and genome rearrangement.

```
CLUSTAL O(1.2.4) multiple sequence alignment

gb|AAG06466.1|+|cprS      MKRGLSLIPIVGGGVTLILAGVLLVYTRMLGDYGETGALYLLSMMMEEEGLYFAQRYQED  60
PYOHw3_10925              MKRGLSLIPIVGGGVTLILAGVLLVYTRMLGDYGETGALYLLSMMMEEEGLYFAQRYQED  60
                          ************************************************************

gb|AAG06466.1|+|cprS      PATPAPDSYYFKGSVGTAGLPPKLREMLDTPPYKSIGAMQLLGNWDDDDEEEDDDAPSDD  120
PYOHw3_10925              PATPAPDSYYFKGSVGTAGLPPKLREMLDTPPYKSIGAMQLLGNWDDNDEEEDDDAPSDD  120
                          ***********************************************:************

gb|AAG06466.1|+|cprS      AYVVVRQPLADGKTLYLYDNDAAGSIDTPLSDAIIDARVRQTWIVTLSVTLPSLAAVGLL  180
PYOHw3_10925              AYVVVRQPLADGKTLYLYDNDAAGSIDTPLSDAIIDARIRQTWIVTLSVTLPSLAAVGLL  180
                          **************************************:*********************

gb|AAG06466.1|+|cprS      VWFIVAPLRKLTRWSMTLDDLAPDSQRPRFNYRELNVLADTLWNSVTRIKDFSQREERFL  240
PYOHw3_10925              VWFIVAPLRKLTRWSMTLDDLAPDSQRPRFNYRELNVLADTLWNSVTRIKDFSQREERFL  240
                          ************************************************************

gb|AAG06466.1|+|cprS      RYASHELRTPLAVIGMNLELLDQPGRAPSPHALQRIRRSALGMQQMTETLLWLSRESGEL  300
PYOHw3_10925              RYASHELRTPLAVIGMNLELLDQPGRAPSPHALQRIRRSALGMQQMTETLLWLSRESGEL  300
                          ************************************************************

gb|AAG06466.1|+|cprS      RDDGHIEVGRLLEELLEEQQALSQRRGLTFHLDVEPHSLPQTRARIIIGNLLRNALQYSD  360
PYOHw3_10925              RDDGHIEVSRLLEELLEEQQALSQRRGLTFHLDVEPHSLPQTRARIIIGNLLRNALQYSD  360
                          ********.***************************************************

gb|AAG06466.1|+|cprS      EGVVEIVVRDRSLLISNPIGAAQGTEESMAFGYGLGLDLVQRLCQKSGWRLHYSSDEQRF  420
PYOHw3_10925              EGVVEIVVRDRSLLISNPIGAAQGTDESMAFGYGLGLDLVQRLCQKSGWRLHYSSDEQRF  420
                          *************************:**********************************

gb|AAG06466.1|+|cprS      RCELLFPATPD   431
PYOHw3_10925              RCELLFPATPD   431
                          ***********
```

Supplementary Figure S2. Alignment of Pseudomonas aeruginosa HW3 Strain with the Reference Strain cprS (gb|AAG06466.1|) using Clustal Omega. The mutations D108N, V159I, and E386D are observed.
